# Prevalence and associated factors of physical inactivity among adults in Northwest Ethiopia: a multicenter study

**DOI:** 10.3389/fpubh.2025.1513600

**Published:** 2025-05-16

**Authors:** Zelalem Tilahun Muche, Awgichew Behaile Teklemariam, Endeshaw Chekol Abebe, Melaku Mekonnen Agidew, Tekile Mengie Ayele, Edgeit Abebe Zewde, Anemut Tilahun Mulu, Gebrehiwot Ayalew Tiruneh, Demewoz Kefale, Deribew Abebaw Abuhay, Milkessa Bayissa Midekssa, Nega Dagnew Baye

**Affiliations:** ^1^Department of Medical Physiology, College of Health Sciences, Debre Tabor University, Debre Tabor, Ethiopia; ^2^Department of Medical Biochemistry, College of Health Sciences, Debre Tabor University, Debre Tabor, Ethiopia; ^3^University of South Australia, Adelaide, SA, Australia; ^4^Department of Pharmacy, College of Health Sciences, Debre Tabor University, Debre Tabor, Ethiopia; ^5^Department of Midwifery (Clinical Midwifery), Debre Tabor University, Debre Tabor, Ethiopia; ^6^Department of Pediatrics and Child Health Nursing, Debre Tabor University, Debre Tabor, Ethiopia; ^7^Department of Sport Science, Sport Science Academy, Haramaya University, Haramaya, Ethiopia; ^8^Department of Human Anatomy, School of Medicine, College of Medicine and Health Science, University of Gondar, Gondar, Ethiopia

**Keywords:** physical inactivity, associated factors, non-communicable diseases, adults, Northwest Ethiopia

## Abstract

**Background:**

Physical inactivity (PI) increases the risk of cardiovascular diseases, cancer, diabetes, cognitive impairment, poor sleep, poor bone health, obesity, economic crises, and premature mortality. Globally, 31% of adults are physically inactive, indicating that this is the major public health issue of this century. Physical inactivity prevalence varies among countries, regions, and cities. In addition, data were scarce in the study area.

**Objective:**

This study identified the prevalence and determinants of PI among adults in Northwest Ethiopia.

**Method:**

A community-based cross-sectional study was conducted from February to March 2024. Participants were recruited via multistage sampling, and the data were collected via WHO stepwise standard questionnaires. Binary and multivariable logistic regressions were used to identify the predictors of physical inactivity.

**Results:**

This study involved 592 participants, with a mean age of 36.2 ± 12.6 years, 56.4% of whom were males. Nearly three-fourths (71.5%) of the respondents had no awareness of physical activity guidelines. The prevalence of PI was 46.1%. Increasing age, being female, being a government or nongovernment employee, having a high level of education, having a high family monthly income, currently smoking, chewing khat, drinking alcohol, being overweight or obese, unawareness of physical activity guidelines, and lacking a plan to do physical activity were associated with PI.

**Conclusion:**

46.1% of adults were physically inactive, highlighting a significant public health concern. Thus, we emphasize improving physical activity by promoting its health benefits, raising awareness about the guidelines, and encouraging adults to have a plan to do physical activity.

## 1 Introduction

Physical activity (PA) is any bodily movement (during leisure time, transport, and work or domestic activities) produced by skeletal muscles that requires energy expenditure. Regular PA improves health and overall well-being. It is vital for the prevention and management of non-communicable diseases (NCDs) (1–3) and increases life expectancy (4). However, many people remain physically inactive (PIA) (3, 5).

Physical inactivity (PI) is one of the leading risk factors for NCDs and mortality (1, 5, 6). It increases the risk of cardiovascular diseases, cancer, diabetes, depression, anxiety, cognitive impairment, poor sleep, poor bone health, obesity, and economic crises (1–6). A meta-analysis revealed that PI increased the risk of breast cancer by 14%, colon cancer by 21%, diabetes by 28%, ischemic heart disease by 25%, and ischemic stroke by 26% (7). PI increases the risk of death by 20%−30% (1), causes more than 1.3 million deaths (4), and costs 27 billion dollars annually (1).

PI is a pandemic and the main health challenge of the twenty-first century (3, 4, 8). The World Health Organization (WHO) reported that 31% (1.8 billion) of adults are PIA globally (1, 8). A study conducted in 2020 revealed that PI is highest in high-income Asian–Pacific countries (48.1%) and lower-middle-income countries (38.2%), and the country-specific prevalence ranged from 2.7% to 66.1% (8). Although the global target is to reduce the PI by 15% by 2030 from the 2010 baseline, it increased by 5% between 2010 and 2022 and will rise to 35% by 2030 (1, 8).

A study performed among 38 Muslim countries worldwide reported that the prevalence of PI was 32.3% (28.6% for non-Arabs and 43.7% for Arabs), indicating that it is higher in the Muslim world (9). The prevalence rates were 66.1% in the United Arab Emirates (8), 43.3% in Nepal (10), 41.1% in Brazil (11), 36.7% in India (12), 36.3% in Malaysia (13), 41.4% in university students in Malaysia (14), 30%−70% in Iran (15), 22.3% in China (16), and 21.6% in Armenia (17).

In Africa, nearly one-fourth (22%) of adults are PIA, and this number is projected to increase. In sub-Saharan Africa, the PI is one of the 10 risk factors for NCD, indicating a wicked problem (3). Several studies have shown that the prevalence of PI is 57.4% in South Africa (18), 52% in Nigeria (19), 49.8% in Umuahia, Nigeria (20), 37.6% in Uganda (21), 53.8% in Khartoum, Sudan (22), 7.7% in Kenya (23), and 2.7% in Malawi (8).

In Ethiopia, a national NCD survey conducted in 2015 revealed that the prevalence of PI is 6% (24). According to the WHO 2022 report on the global status of PA: country profile, 12% of men and 20% of women in Ethiopia are PIA (25). However, other individual studies on different cities in Ethiopia have shown a higher prevalence, ranging from 29.5% to 65.6% (26–33). In addition, the PI among patients with chronic conditions in Ethiopia ranges from 30% to 68% (34–36).

Moreover, many studies have indicated that sociodemographic factors (age, sex, occupation, educational status, marital status, residence, and monthly income), behavioral factors (khat chewing and alcohol consumption), health factors (diabetes, heart disease, cancer, chronic respiratory diseases, and hypertension), overweight or obesity, and having no information about PA guidelines are associated with PI (13, 14, 16, 17, 29–34).

The prevalence of PI in Ethiopia is high and increasing. Its prevalence varies across regions and cities. Many studies conducted in Ethiopia have indicated that the magnitude of PI among adults is 45.1% in Dire Dawa City (32), 45.5% in Harar City (30), 44.1% in Wolaita Sodo City (28), Haramaya University (49.1%) (27), and Southwest Ethiopia (61.2%) (29). Two studies done in Northwest Ethiopia revealed that 65.6% of **older** adults in Gondar City (33) and 37.9% of adults in Bahir Dar city are PIA (31). Moreover, there are limited data in our study area. Therefore, this study assessed the prevalence and associated factors of PI among adults in towns and City of South Gondar Zone, Northwest Ethiopia.

## 2 Materials and methods

### 2.1 Study setting, design, and period

A community-based cross-sectional study was conducted among adults residing in South Gondar Zone, Amhara regional state, Northwest Ethiopia (https://www.mindat.org/loc-406543.html) from March to April 2024. This zone has 14 district towns and one metropolitan city (Debre Tabor) that contains three sub cities or woredas. The city is located 665 km from Addis Ababa and 100 km from Bahir Dar city. According to the 2007 census conducted by the Central Statistical Agency of Ethiopia, this zone has a total population of 2,051,738 and an area of 14,095.19 square kilometers. It has one tertiary hospital and nine primary hospitals.

### 2.2 Population

The source of population were all adults ≥18 years old who lived in towns and cities of the South Gondar Zone in Northwest Ethiopia. The study population were all adults ≥18 years old who lived in the study area, and were available during the data collection. Adults aged ≥18 years old and who lived for ≥6 months in the study area were included in the study, whereas pregnant women, lactating women, severely sick individuals, and disabled people were excluded from the study.

### 2.3 Sample size determination and sampling procedure

The sample size was determined via a single population proportion formula, where the prevalence of PI among adults in Dire Dawa City (the capital city of Dire Dawa in East Ethiopia) was 45.1% (32).


(1) $$\begin{array}{l} n\;=\;\frac{{\left(Z\alpha /2\right)}^{2}p\left(1-p\right)}{d^{2}}\;=\\ \;\frac{{\left(1.96\right)}^{2}\times 0.452\left(1-0.452\right)}{{\left(0.05\right)}^{2}}=380.47\approx \;381. \end{array}$$

where *n* = sample size, *z* = standard normal variable at the 95% confidence level (1.96), *p* = the prevalence of PI in Dire Dawa City (0.451), and *d* = margin of error (0.05).

Considering the design effect 1.5, *n* = 381 × 1.5 = 571.5 ≈ 572, then adjusted for non-response rate (5%) and *n* = 572 + 572 × 5/100 = 600.6 ≈ **601**.

**Figure 1:**
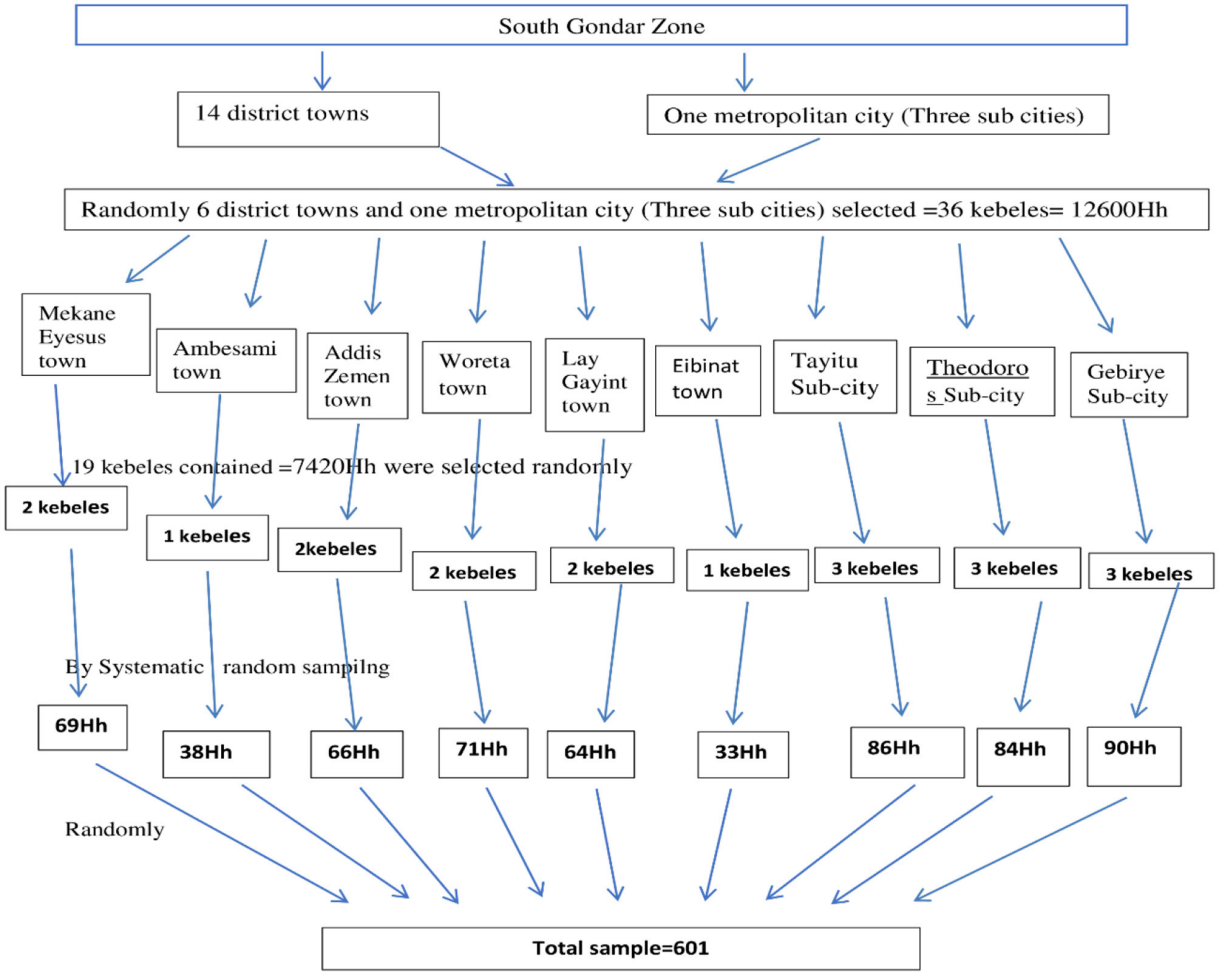
Sampling procedure for the selection of participants in the South Gondar Zone, Northwest Ethiopia, 2024 (*n* = 592). Hh, House hold.

Multistage sampling was employed to select the participants. Among 14 towns and one metropolitan city that contains three sub cities, 6 towns and one metropolitan city that contains 36 kebeles (lowest level of local government in Ethiopia) with 12,600 households (Hh) were randomly selected. From these, we randomly selected 19 kebeles, which contains 7,420 Hh. Of these we selected 601 Hh proportionally via systematic random sampling from each kebele. Finally, participants were randomly selected from each Hh (Figure 1).

### 2.4 Study variables

Physical inactivity was the dependent variable. Sociodemographic factors (age, sex, occupation, educational status, marital status, and monthly income), behavioral factors (smoking, khat chewing, and alcohol consumption), overweight or obesity, central obesity, presence of community PA program, having a plan to do PA, time for PA, and having no information about PA guidelines were independent variables.

### 2.5 Operational and term definitions

**BMI** (**kg/m**^**2**^) was classified into the following categories: underweight (< 18.5 kg/m^2^), normal (18.5–24.9 kg/m^2^), overweight (25.0–29.9 kg/m^2^), or obese (≥30.0 kg/m^2^) (5).

**Central obesity:** Male participants with a waist circumference of > 94 cm and female participants with a waist circumference of > 80 cm were classified as having central obesity (5).

**High physical activity**: a person fulfilling any of the following criteria:

- Vigorous-intensity activity on at least 3 days, achieving a minimum of at least 1,500 metabolic equivalents (MET)-minutes per week or,
- Seven or more days of any combination of walking, moderate- or vigorous-intensity activities achieve a minimum of at least 3,000 MET-minutes per week (5).

**Moderate physical activity**: a person not meeting the criteria for the “high” category but meet any of the following criteria:

- Three or more days of vigorous-intensity activity of at least 20 min per day or,
- 5 or more days of moderate-intensity activity or walking for at least 30 minutes per day or,
- Five or more days of any combination of walking, moderate- or vigorous-intensity activities achieve a minimum of at least 600 MET-minutes per week (5).

**Low physical activity**: not meeting any of the abovementioned criteria under moderate- or high-intensity physical activities (5).

**Physically Active:** A person who performs at least 150 min of moderate-intensity physical activity or 75 min of vigorous-intensity physical activity (fulfills the WHO recommendation on the PA of adults) (5). **Physically Inactive:** A person who does not perform at least 150 min of moderate-intensity physical activity or 75 min of vigorous-intensity PA (does not fulfill the WHO recommendation on the PA of adults) (5). **Past smoker**: a person with a history of use of any tobacco product (5). **Current smoker**: an adult who smoked a cigarette at least once in the last 30 days before the data collection time (5).

**Past alcohol drinker**: a person with a history of any type of alcohol use (5). **Current alcohol drinker**: a person with a history of any type of alcohol use in the past 30 days (5). **Past khat chewer:** a person with a history of use of any tobacco product (5). **Current khat chewer**: a person with a history of chewing khat in the past 30 days (5).

### 2.6 Data collection and measurement

Data were collected, and parameters were measured via the WHO stepwise approach to NCD risk factor surveillance (instrument v.3.2) standard questionnaires and measurement principles (5). All sociodemographic, health-related, and behavioral data were collected through face-to-face interviews conducted by six trained nurses over a period of 30 days, and the information was documented by the data collectors. Following the collection of this data, anthropometric measurements were taken. Height was measured using a stadiometer to the nearest 0.1 cm with the subjects positioned on the Frankfurt plane without shoes or a cap. The back of the head, shoulder blades, buttocks, and heels touch the stadiometer. Weight was measured via a digital glass weight scale to the nearest 0.1 kg, with the subjects wearing light clothes and shoes removed. Waist circumference was measured at the midpoint between the lowest costal margin at the midclavicular line and the anterior superior iliac spine via fixed tension tape. All anthropometric measurements were performed in triplicate, and the average value was used. BMI was calculated as weight in kilograms (kg) divided by height in meters squared (kg/m^2^). Physical activity questions, which focus on three domains (work, transport, and recreation) were gathered using the WHO stepwise approach to NCD risk factor surveillance, part five, Section 2 (question-by-question guide, version 3.2, pages 5-2-10 to 5-2-11) (5). We utilized the core aspects of the PA questions to obtain essential information about the types of activities (both vigorous and moderate-intensity), the number of days each type of activity is performed per week, and the duration of each activity conducted during the week.

### 2.7 Data processing and statistical analysis

The data were checked for completeness, cleaned, entered into Epi Info version 7, and exported to SPSS version 25. The categorical and continuous data were summarized by frequency and percentage and means ± standard deviations, respectively, and were presented in tables and graphs. Univariable and multivariable logistic regression were performed to identify the predictors. During binary logistic regression, variables with *P*-values < 0.25 were entered into a multivariable logistic regression. The model's fitness was determined via the Hosmer–Lemeshow goodness-of-fit statistic. Homogeneity of variances, collinearity, and outliers were checked. The degree of association was assessed via the crude odds ratio (COR) and adjusted odds ratio (AOR) with their respective 95% CIs, and a *P*-value < 0.05 indicated statistical significance.

### 2.8 Data quality control and management

We used the WHO stepwise standard questionnaires, which were translated from English into the local Amharic language by English teachers at Debre Tabor University. A pretest was undertaken in 5% of the study population at Woreta town to check the completeness of the questionnaire, clarity of language, and consistency. The completeness of the data was checked during data collection, entry, and analysis.

## 3 Results

### 3.1 Sociodemographic characteristics of the participants

A total of 592 respondents participated, with a response rate of 98.5%. The mean age of the respondents was 36.2 ± 12.6 years, and the majority (51%) were 30–44 years. The majority were male (56.4%), married (67.1%), Orthodox Christian (85.5%), and Amhara ethnic (92%). 25.1% of the participants had no formal education (Table 1).

**Table 1:** Sociodemographic characteristics of adults aged 18–64 years in Northwest Ethiopia, 2024 (*n* = 592).

**Variables**	**Category**	**Frequency**	**Percent (%)**
Sex	Male	334	56.4
	Female	258	43.6
Mean age (years)		36.2 ± 12.6	
Age category (years)	18–29	130	22.0
	30–44	302	51.0
	45–64	160	27.0
Marital status	Single	143	24.2
	Married	397	67.1
	Widowed or separated	51	8.7
Religion	Orthodox Christian	506	85.5
	Muslim	86	14.5
Ethnicity	Amhara	545	92.1
	Others	47	7.9
Level of education	No formal education	149	25.1
	Primary and preparatory school	233	39.4
	Tertiary education	210	35.5
Occupation	Laborer	72	12.2
	Government and NGO employee	153	25.8
	Retired	14	2.4
	Self-employee	190	32.1
	House wife	80	13.5
	Unemployed	48	8.1
	Student	35	5.9
Monthly family income (*Ethiopian birr*)	< 5,000 ETB	270	45.6
	5,000–10,000 ETB	189	31.9
	≥10,000 ETB	133	22.2
Family size (number)	≤ 4	364	61.5
	≥5	228	38.5

### 3.2 Behavioral and anthropometric characteristics of the respondents

This study revealed that 65 (10.5%), 118 (19.9%), and 501 (84.6%) of the participants were current smokers, khat chewers, and alcohol drinkers, respectively. The majority of the participants had central obesity (35.1%), had no information about PA guidelines (71.5%), and had time for PA (66.9%) (Table 2).

**Table 2:** Behavioral and anthropometric characteristics of adults aged 18–64 years in Northwest Ethiopia, 2024 (*n* = 592).

**Variables**	**Category**	**Frequency**	**Percent (%)**
Smoking status	Never	493	83.3
	Past smoker	37	6.2
	Current smoker	62	10.5
Khat chewing status	Never chew	428	72.3
	Past chewer	46	6.8
	Current chewer	118	19.9
Alcohol drinking status	Never drunk	58	9.8
	Past drinker	33	5.6
	Current drinker	501	84.6
Mean height (m)		1.65 ± 1.6	
Mean weight (kg)		57.5 ± 11.3	
BMI (kg/m^2^)	Underweight	70	11.8
	Normal	354	59.8
	Overweight	150	25.3
	Obese	18	3.1
	< 25 kg/m^2^	424	71.6
	≥25 kg/m^2^	168	28.4
Central obesity (cm)	Yes	208	35.1
	No	384	64.9
Do you have information about PA guideline's	Yes	169	28.5
	No	423	71.5
Do you have time for PA	Yes	396	66.9
	No	196	33.1
Presence of community PA program	Yes	52	8.8
	No	540	91.2
Having a plan to do PA	Yes	113	19.1
	No	479	80.9

BMI, body mass index; kg, kilogram; PA, physical activity.

### 3.3 Health-related characteristics of the participants

Among all the participants, 79 (13.3%), 48 (8.1%), and 22 (3.7%) had hypertension, diabetes, and chronic kidney disease, respectively (Table 3).

**Table 3:** Health-related characteristics of adults aged 18–64 years in Northwest Ethiopia, 2024 (*n* = 592).

**Variables**	**Category**	**Frequency**	**Percent (%)**
History of hypertension	Yes	79	13.3
	No	513	86.7
History of diabetes	Yes	48	8.1
	No	544	91.9
History of cancer	Yes	12	2.0
	No	580	98
History of heart disease	Yes	16	2.7
	No	576	97.3
History of chronic respiratory disease	Yes	18	3.0
	No	574	97
History of chronic liver disease	Yes	15	2.5
	No	577	98.5
History of chronic kidney disease	Yes	22	3.7
	No	570	96.3

### 3.4 Prevalence of PI among respondents

**Figure 2:**
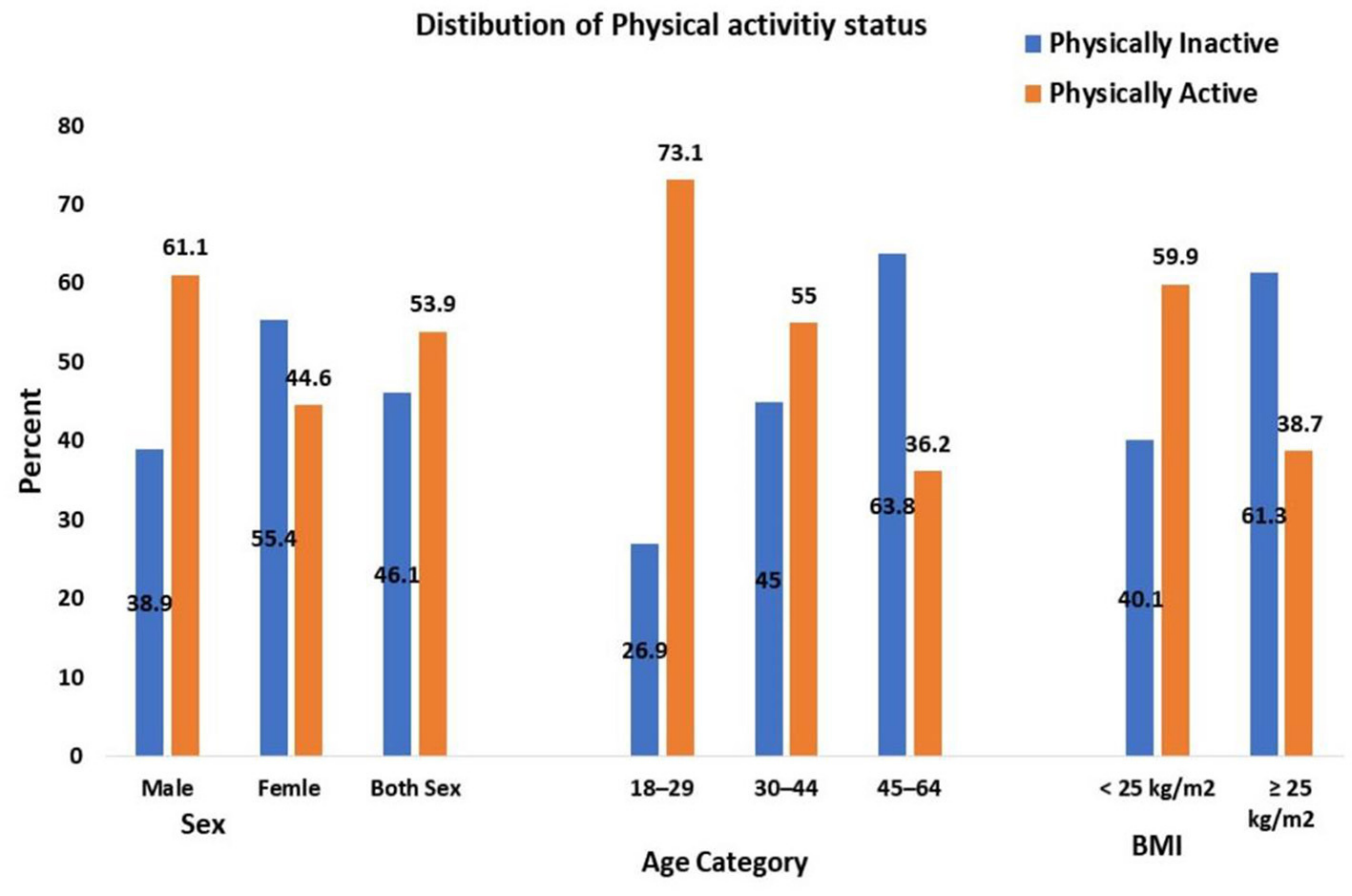
Physical activity status of adults aged 18–64 years in Northwest Ethiopia, 2024 (*n* = 592). BMI, Body mass index, kg/m^2^, kilogram per meter square, age expressed in years.

This study revealed that 98 (16.6%), 221 (37.3%), and 273 (46.1%) of the participants had high, moderate, and low levels of PA, respectively. The prevalence of PI was 273 (46.1%) (Figure 2).

### 3.5 Factors associated with PI

The odds of PI among participants aged 30–44 and 45–64 years were four and seven times greater than those among participants aged 18–29 years, respectively (adjusted odds ratio (AOR) = 4.2, 95% CI: 2.89, 10,34; AOR = 7.1, 95% CI: 4.53, 12.16). Women were 3.7 times more likely to have PI than men (AOR = 3.7, 95% CI: 2.48, 10.82). Those who have attended tertiary education, government and NGO employees and those with a family monthly income ≥10,000 Ethiopian Birr (ETB) were 4.75, 3.1, and 3.7 times more likely to have PI than single, those who had no formal education, laborers, and those with a family monthly income < 5,000 ETB, respectively. Current smokers, khat chewers, and alcohol drinkers were 6.26, 5.79, and 8.05 times more likely to have PI than non-smokers, never chewed khat, and non-drinkers, respectively. Respondents who had no information about PA guidelines and lacked plans to perform PA were five and seven times more likely to have PI than their counterparts (Table 4).

**Table 4:** Binary and multivariable logistic regression analysis of factors associated with physical inactivity among adults aged 18–64 years in Northwest Ethiopia, 2024 (*n* = 592).

**Variables**	**PI**			
	**No (%)**	**Yes (%)**	**COR (95% CI)**	**AOR (95% CI)**
**Age category (years)**				
18–29	95 (73.10)	35 (26.90)	1	1
30–44	166 (55.00)	136 (45.00)	5.93 (4.17, 13.05)^**^	4.20 (2.89, 10,34)^*^
45–64	58(36.20)	102 (63.80)	14.22 (10.20, 21.70)^***^	7.10 (4.53, 12.16)^**^
**Sex**				
Male	204 (61.10)	130 (38.90)	1	1
Female	115 (44.60)	143 (55.40)	2.19 (1.64, 8.49)^***^	3.70 (2.48, 10.82)^*^
**Marital status**				
Single	131 (91.60)	12 (8.40)	1	1
Married	258 (65.00)	139 (35.00)	5.88 (4.56, 11.02)^***^	3.21 (0.84, 12.10)
Widowed or Separated	29 (56.90)	22 (43.10)	8.28 (7.59, 16.42)^**^	9.03 (0.90, 17.71)
**Level of education**				
No education	116 (77.90)	33 (22.10)	1	1
1 and 2 school	142 (60.90)	91 (39.10)	2.32 (1.65, 6.94)^**^	3.38 (0.79, 11.05)
College and above	111 (52.90)	99 (47.10)	3.13 (2.13, 5.62)^***^	4.75 (2.25, 16.48)^**^
**Occupation**				
Laborer	63 (87.50)	9 (12.50)	1	1
Government and NGO	96 (62.70)	57 (37.30)	4.15 (2.10, 14.57)^**^	3.14 (1.43, 10.74)^**^
Self-employee	116 (61.90)	74 (38.90)	4.46 (2.35, 16.1)^***^	4.60 (0.69, 15.32)
House wife	56 (70.00)	24 (30.00)	3.00 (1.43, 6.17)^**^	5.00 (0.87, 12.45)
Others^†^	72 (74.20)	25 (25.80)	2.43 (1.07, 10.22)^*^	2.63 (0.50, 8.90)
**Family monthly income (ETB)**				
< 5,000 ETB	202 (74.80)	68 (25.20)	1	1
5,000–10,000 ETB	116 (61.40)	73 (38.60)	1.87 (0.35, 8.94)	3.38 (0.81, 18.05)
≥10,000 ETB	69 (51.90)	64 (48.10)	2.76 (2.13,5.62)^***^	3.70 (2.25,16.48)^**^
**BMI (kg/m** ^2^ **)**				
< 25 kg/m^2^	254 (59.90)	170 (40.10)	1	1
≥25 kg/m^2^	65 (38.70)	103 (61.30)	1.36 (1.0, 6.39)^**^	2.54 (1.9, 9.67)^**^
**Central obesity(cm)**				
No	314 (81.80)	70 (18.20)	1	1
Yes	89 (47.30)	99 (52.70)	4.98 (3.59, 7.73)^*^	3.60 (0.9, 23.4)
**Having information about physical activity guideline's**				
Yes	134 (79.30)	35 (20.70)	1	1
No	253 (59.8)	170 (40.20)	2.57 (2.03,8.23)^**^	5.56 (2.41,9.02)^***^
**Plan to do physical activity**				
No	339 (70.80)	140 (29.20)	2.84 (1.9,10.56)^***^	7.80 (5.37, 19.50)^**^
Yes	52 (46.00)	61 (54.00)	1	1
**Do you have time for physical activity**				
No	311 (78.50)	85 (21.50)	1	1
Yes	138 (70.40)	58 (29.60)	1.53 (1.01, 5.89)^*^	2.70 (1.32,8.55)
**Presence of community physical activity program**				
No	378 (70)	162 (30.00)	1	1
Yes	33 (59.60)	19 (39.40)	1.34 (1.0, 6.2)^*^	2.3 (0.79,5.48)
**Smoking status**				
Never smoke	379 (76.90)	114 (23.1)	1	1
Past smoker	20 (54.10)	17 (45.9)	2.83 (2.12, 6.04)^***^	3.14 (0.66, 7.83)
Current smoker	22 (35.50)	40 (64.5)	6.04 (3.39,13.2)^***^	6.26 (2.93,16.48)^*^
**Khat chewing status**				
Never chew khat	338 (79.00)	90 (21.00)	1	1
Past chewer	19 (41.30)	27 (58.70)	5.34 (3.23, 11.34)^***^	4.67 (0.87, 15.61)
Current chewer	55 (46.4)	63 (53.40)	4.30 (2.35, 5.87)^***^	5.79 (1.60, 8.96)^*^
**Alcohol drinking status**				
Never drink	45 (77.6)	13 (22.4)	1	1
Past drunker	21 (63.6)	12 (36.4)	1.97 (1.01, 7.93)^*^	2.13 (0.76, 12.73)
Current drinker	348 (69.5)	153 (30.5)	2.52 (2.01, 8.46)^**^	8.05 (2.37, 18.1)^**^

ETB, Ethiopian birr; BMI, body mass index; PA, physical activity; COR, crude odds ratio; AOR, adjusted odds ratio; CI, confidence interval; NGO; non-governmental organization.

^**†**^Retired + unemployed + student.

^***^p-value < 0.001,^**^p-value < 0.01,^*^p-value < 0.05.

## 3 Discussion

The current study revealed that the proportion of PI among adults was 46.1%. This finding is consistent with other studies that reported 45.5% in Harar City, Ethiopia (30); 45.1% in Dire Dawa City, Ethiopia (32); and 44.1% in Wolaita Sodo City, Ethiopia (28).

Our finding is lower than the studies conducted in the United Arab Emirates (66.1%) (8), the United Kingdom (64%) (37), South Africa (57.4%) (18), Khartoum, Sudan (22), Nigeria (52%) (19), Umuahia, Nigeria (49.8%) (20), Iran (30–70%) (15), Southwest Ethiopia (61.2%) (29), Haramaya University, Ethiopia (49.1%) (27), and Gondar City, Ethiopia (65.6%) (33). This inconsistency could be due to differences in the study area, study period, sociodemographic characteristics, behavioral and anthropometric characteristics, and health-related factors. For example, in the United Arab Emirates (8) and the United Kingdom (37), there may be high income and high urbanization, and people may use vehicles for transport, which can increase the odds of PI (12, 14, 16, 30). One study reported that the PI exceeded 50% in the United Arab Emirates, Kuwait, Cuba, Lebanon, South Korea, Panama, Qatar, Iraq, Portugal, and Saudi Arabia (8). Another explanation is that participant occupation differences between studies can cause the above variation (27).

However, the prevalence of PI in this study is higher than other studies that showed the magnitude of PI was 43.3% in Nepal (10), 41.1% in Brazil (11), 36.7% in India (12), 36.3% in Malaysia (13), 22.3% in China (16), 21.6% in Armenia (17), 37.6% in Uganda (21), 7.7% in Kenya (23), 2.7% in Malawi (8), 6% to 20% in Ethiopia (24, 25), 29.5% in Bale zone towns, Ethiopia (26), 37.9% in Bahir Dar City, Ethiopia (31). This difference could be due to differences in the study area, study period, sociodemographic characteristics, behavioral and anthropometric characteristics, and health-related factors. For example, the Ethiopia National NCD survey revealed that the PI was 6%; this survey was conducted 9 years ago, and it included both urban and rural residents (24). The PI varies across countries, regions, and cities (1, 3, 8). In the present study, almost five out of every 10 adults were PIA. This finding suggests the need to promote PA, which in turn requires a collaborative public health response targeting older, female, and employed adults (12, 26, 32).

The present study revealed that the likelihood of PI increases as age increases. The odds of PI among those aged 30–44 years and 45–64 years were 4.2 and 7.1 times greater than those aged 18–29 years, respectively. This finding is consistent with several previous studies (10, 29, 30, 33, 34). This can be explained by aging, which causes a reduction in muscle strength, flexibility, agility, changes in body fat percentage, and endurance, which impacts PA (38). Age increases the odds of PI, which emphasizes the need for community-based PA programs targeting older adults (12, 29, 33, 38).

In this study, being female increased the odds of PI by 3.7 times. This finding is similar to the previous studies showing that being female increases the odds of PI (10, 13, 17, 29, 30, 32, 34). This can be explained by females spending more time engaging in sedentary behavior than males do, either due to cultural or other factors. One study revealed that the global prevalence of PI in 2022 was 5% higher in females than in males. However, this difference can be >10% in one-third of countries and >20% in six countries such as Afghanistan (8). This finding warrants encouraging women to do PA by avoiding gender role variations and increasing the availability of physical infrastructures suitable for women (26, 29).

Our study revealed that participants who have attended tertiary education were 4.75 times more likely to have PI than those who had no formal education. This may be because as the level of education increases, there will be more chances to be employed in organizations that need sedentary behavior to do their work. This is supported by many other studies (10, 24, 29, 33). However, other studies did not report such an association (27, 32). The present study showed that being a government or NGO employee increases the odds of PI by 3.1 times as compared to a laborer. This result is similar to studies that showed that being a government employee (10, 16, 22, 32) and being a non-manual worker (27) increases the odds of PI. Conversely, a study performed in southeast Ethiopia showed that being unemployed increases the odds of PI (26). The participants who had a family monthly income ≥10,000 **ETB** were 3.7 times more likely to have PI than those who had < 5,000 ETB (32). This finding is consistent with several studies that indicated that high income increases the likelihood of PI (12, 14, 16, 18, 20, 30). Generally, as the level of education and income increase, sedentary behavior increases, which can increase the odds of PI. This highlights the need to increase the PA by inspiring the adults to have a plan to do PA (20, 29).

In this study, overweight or obesity increased the likelihood of PI by 2.54 times. This finding is supported by other studies that showed that overweight or obesity increases the odds of PI (10, 20–22, 26, 27). The results of our study indicated that respondents who had no information about PA guidelines were five times more likely to have PI than their counterparts. This finding is consistent with another study conducted in Harar City, Ethiopia, which revealed that having no information about PA guidelines increases the likelihood of PI by 3.5 times (30, 39). This can be explained by a study conducted in Ethiopia that showed that only 27% of participants had good knowledge of the PA guidelines (40). Some studies revealed that increasing unawareness about the PA guidelines decreases the likelihood of PI (29, 30, 39, 40).

In our finding having time is not significantly associated with PI.

The present findings revealed that current khat chewers and alcohol drinkers were more than five and eight times more likely to have PIs than never chewed khat and non-drinkers, respectively. This finding coincides with studies that reported that khat chewing (27) and alcohol consumption (20, 27) increase the odds of PI. This could be due to sedentary behavior during khat chewing and alcohol consumption and the consequences of these habits. Hence avoiding smoking and alcohol drinking may increase PA.

## 4 Strength and limitations of the study

This study stands out for its novelty as the first of its kind in the area, offering valuable insights, a baseline data for informing future research and public health intervention, we used standard tools, and questionnaires were pretested. However, the study has potential limitations. First, the study design was a cross-sectional study, which does not explain the cause-effect relationship. Second, the study was done in a specific study area (Northwest Ethiopia) rather than at the national level, making it difficult to generalize the findings to other regions. Third recall bias might be introduced in the study as we rely on self-reporting data. Hence, longitudinal studies should be done to understand the dynamics and the causation of the observed factors.

## 5 Conclusion

This study revealed a high prevalence of PI among adults in Northwest Ethiopia, indicating that it is an epidemic in the community. Being a government or non-government employee, having a high level of education, having a high family monthly income, current smoking, khat chewing, alcohol drinking, being overweight or obese, having no information about PA guidelines, and lacking a plan to perform PA were associated with PI. As a result of high PI, the majority of adults are at a greater risk of developing NCDs, other health problems, and premature death. Consequently, community-based interventions are essential to increase PA. Priority should be given to specific groups, including older adults, women, government and non-government employees, those who have attended tertiary education, those with high income, smokers, khat chewers, and alcohol drinkers. Besides, we should emphasize increasing PA by increasing awareness about PA guidelines via health education and encouraging adults to have a plan to do PA.

## Data Availability

The original contributions presented in the study are included in the article/supplementary material, further inquiries can be directed to the corresponding author.
